# Emergence of drug resistant mutations after single dose nevirapine exposure in HIV-1 infected pregnant women in south India

**Published:** 2010-11

**Authors:** Lakshmi Rajesh, K. Ramesh, Luke Elizabeth Hanna, P.R. Narayanan, Soumya Swaminathan

**Affiliations:** *Tuberculosis Research Centre (ICMR), Chennai, India*

**Keywords:** Drug resistance, HIV, nevirapine, pregnancy

## Abstract

**Background & objectives:**

Resistance to nevirapine (NVP) has been described with single dose preventive regimens in other populations. Our aim was to study the pattern and prevalence of HIV drug resistance (DR) at baseline (during pregnancy) and after delivery among antenatal women exposed to single dose NVP for prevention of parent to child transmission (PPTCT).

**Methods:**

HIV-infected, ART-naive primigravidae between 18-25 years of age, attending government antenatal clinics in Chennai, Vellore or Madurai were recruited. Drug resistance testing was carried out during pregnancy and after Sd-NVP treatment (one month after delivery) by Viroseq sequencing. HIV-1 testing by DNA PCR was done in newborns at 30 days.

**Results:**

Thirty one women were enrolled but only twenty six plasma specimens were analyzable (24 paired and two postnatal only). No major mutations were observed in any drug class at baseline though many polymorphisms were observed in both the reverse transcriptase and protease genes. Mutations to non-nucleoside reverse transcriptase inhibitors (NNRTI) were observed post-delivery in 33 per cent of women who were treated with Sd-NVP. None of the infants were HIV-positive.

**Interpretation & conclusions:**

Among pregnant ART-naïve women, baseline HIV drug resistance was not observed. A high rate of development of NNRTI class resistance among women treated with single-dose NVP was observed. Our results emphasize the need to implement more effective PPTCT regimens, minimizing emergence of drug resistance and thereby preserving long-term treatment options for HIV-infected women in India.

In India, HIV prevalence among pregnant women is reported to be 0.3 per cent^1^. HIV transmission from mother to child can occur *in utero*, intrapartum (during labour) or postpartum (through breastfeeding). In the absence of any interventions, about 25-30 per cent of infants will be infected with HIV. Several regimens have been tested for prevention of parent-to-child transmission (PPTCT), including nevirapine (NVP) given as a single dose to the mother at the time of delivery followed by a single dose to the infant within 72 h (Sd-NVP)^2^.

Resistance to nevirapine has been described after use of single drug preventive treatment, which is why triple drug therapy is recommended as the preferred option for treatment^3^,^4^. While the prevalence of resistance mutations decreases with time after exposure to Sd-NVP, there is some evidence that women with pre-existing mutations as well as those exposed to Sd-NVP but without mutations respond less well to antiretroviral treatment^5^. Emergence of antiviral drug resistant mutations (DRM) among Indian women who had received Sd-NVP regimen for PPTCT is not described. Our aim was to study the presence and pattern of drug resistance (DR) at baseline and after delivery among antenatal women exposed to Sd-NVP and determine the HIV status of their infants.

## Material & Methods

Consecutive HIV-1 infected pregnant women attending government maternity hospitals in Chennai, Madurai and Vellore between July 2007 and March 2008 who were primigravidae, 18-24 yr old and had no prior history of antiretroviral treatment were recruited for the study. Pregnant women who came to the hospital for the first time in labour, those with a previous history of ART, those attending the antenatal clinic for obstetric consultation but likely to go elsewhere for delivery and those who had any cognitive dysfunction were excluded.

The clinics followed NACO guidelines for PPTCT and administered a single dose of nevirapine to the mother at the time of delivery and one dose to the infant within 72 h after birth. These government clinics catered to women from the lower socio-economic strata and breast feeding was followed by >90 per cent of them. After obtaining written informed consent, 5 ml blood was drawn during the 2^nd^ or 3^rd^ trimester, for genotypic testing. Women (and their infants) were seen and blood collected during the postnatal visit one month after delivery (after confirmation that they had received Sd-NVP). DNA PCR was performed with Roche Amplicor HIV 1 DNA, v 1.5 kit (Roche Diagnostic Systems Inc., Brandburg, NJ, USA) following manufacturers instructions, to determine the newborn’s HIV status. If the child was found to be HIV positive, then plasma was screened for the presence of DRMs. Genotypic drug resistance testing was performed for reverse transcriptase (RT) and protease (PR) genes using the ViroSeq genotyping kit (Celera Diagnostics, USA) on an Avant 3100 Genetic Analyzer (Applied Biosystems, USA) using standard protocols^6^. If RNA could not be amplified, viral load was estimated in plasma using COBAS Amplicor HIV-1 monitor test kit (Roche Diagnostics, Germany). The interpretation of the observed mutations was performed using the Stanford Drug resistance database^7^. There are approximately 75 HIV drug resistance-associated mutations documented worldwide including 17 nucleoside reverse transcriptase inhibitors (NRTI), 18 nonNRTI (NNRTI), and 40 protease inhibitors (PI)-associated mutations^8^. The mutations that are commonly observed in the Indian studies^9^–^11^ are as follows:-

NRTI related mutations - M184V, L74V followed by the thymidine analog mutations (M41L, K219E, D67N, T215S).

NNRTI related mutations - Y181C, K103N, V106M, G190A, K101E, & Y188C and

PI related mutations - M46I, I47A, V82I & L90M.

The study protocol was approved by the Institutional Ethics committee of the Tuberculosis Research Centre (TRC), Chennai.

## Results

Thirty one HIV-1 infected pregnant women were recruited; however the analysis is restricted to 26 samples that were available for DR testing, 24 from twelve women at both time points and 2 samples postnatally only (after Sd-NVP treatment). Due to the fact that many women delivered in cities and then returned to their native place, follow-up was challenging. Most of the deliveries occurred by Caesarian section. Of these 26 samples, six (4 antenatal and 2 after Sd-NVP treatment) could not be amplified while successful amplification of viral RNA was obtained in the remaining 20 samples. Viral load testing of the six unamplified samples revealed undetectable viral load (< 400 copies/ml) in all of them, suggesting that they may have been on antiretroviral treatment. Hence, results were available for 8 women antenatally and 12 women at 1 month after delivery. With respect to the reverse transcriptase (RT) gene, polymorphisms were commonly observed at codons 35, 36, 39, 48, 60, 121, 135, 162, 173, 177, 200, 207, 214, 245, 286, 291, 292, 293 and 294. In the protease (PR) gene secondary (minor) mutations at positions 12, 14, 15, 19, 36, 37, 41, 63, 69, 79, 89 and 93 were observed most commonly. Complete concordance was seen in RT and PR gene polymorphisms in sequences obtained from paired samples of the same patient. No major DRMs to NRTIs or PIs were observed at either time point.

There were no major NNRTI mutations observed in any of the patients at baseline. DR mutations conferring resistance to NNRTI were observed in 4 (of 12) patients after treatment with Sd-NVP, (33%, 95% CI 22-44%). The mutations observed were Y181C, K103N and Y188C conferring resistance to both NVP and efavirenz (EFZ) (Table). Two women had the K103N mutation, one had Y181C mutation and one had the Y188C mutation together with the K103N mutation. None of the 15 infants (one mother delivered twins) born to these mothers was found to be HIV-1 infected by DNA PCR at 1 month. The breast feeding status of the infants was not known.

**Table:** NNRTI drug resistant mutations in the rt gene at baseline and follow up

	NNRTI mutations		
ART regimen	K103 N	Y181 C	Y188C
Baseline (N=8)	Nil	Nil	Nil
Post Sd-NVP (N=12)	3	1	1*

* 1 woman had more than one mutation at follow up

## Discussion

In this pilot study of women treated with Sd-NVP, one-third had a detectable NNRTI resistance mutation at 1 month after delivery. The major resistance mutations observed were K103N and Y181C which confer cross-resistance to all the NNRTIs. NNRTI mutations were observed only after treatment with Sd-NVP as none of the women had evidence of drug resistance when tested during pregnancy. While polymorphisms (minor mutations) were common, no major mutations against NRTI or protease inhibitors were observed either before or after treatment. The selection of young, primigravidae pregnant women for this study ensured that the majority were probably recently infected and had no exposure to antiviral drugs previously. Absence of baseline drug resistance in this group implies low rate of transmission of resistant viruses in the general population. None of the infants tested was found to be HIV-infected, though the numbers were small. However, DNA PCR testing at 4 wk would detect only the antenatal and intrapartum infections and late postnatal transmission through breast feeding would still be possible.

Previous studies on HIV-infected pregnant women have reported variable rates of emergence of drug resistance after prophylactic treatment. Resistance to nevirapine after single-dose treatment has been reported in 19 per cent of women with subtype A, 36 per cent with subtype D and 69 per cent with subtype C virus, which is the main subtype in India^12^. A meta-analysis including all these studies estimated the pooled prevalence of NVP resistance after single dose NVP to be 35.7 per cent and it was shown that this could be substantially reduced by adding short course post-partum ARV therapy to standard prophylaxis^13^,^14^. Other studies have reported NVP resistance among 33-38 per cent of infants with HIV when both the infants and their mothers were exposed to Sd-NVP^13^,^15^. In most cases, infants with resistance to NVP were noted to be infected at birth, suggesting that the resistance mutations were selected *de novo* among the infants when their actively replicating virus was exposed to NVP rather than being transmitted from their mother^16^.

Our findings have important clinical and programmatic implications both for PPTCT and for future treatment of both mothers and their infants in India. A study in Thailand suggested that maximal viral suppression might not be achieved in women who received Sd-NVP and were later started on NVP-based antiretroviral therapy (ART). However, the length of time between exposure to Sd-NVP and initiating NNRTI-based ART appears to be an important factor affecting treatment response, with mutations having a diminished effect when the gap between Sd-NVP and ART initiation was longer^5^. Another recent study made similar observations in children^17^. However, the full implications of archived mutations are as yet unknown. A randomized trial in South Africa reported that administration of the drugs AZT and 3TC during labour along with Sd-NVP followed by AZT and 3TC for four to seven days post-partum reduced the rate of development of resistance to NVP from 60 per cent to about 10 per cent^18^. As NVP has a long half-life and drug levels persist for up to three weeks in plasma, it is expected that giving dual NRTI regimens for a period after the women receive Sd-NVP would suppress viral replication and decrease the risk of developing resistance. For these reasons, the 2009 revised WHO guidelines for PPTCT recommend antiretroviral treatment for all pregnant women with symptoms or CD4 counts < 350 cells/mm^3^ and a regimen of zidovudine followed by Sd-NVP or triple drug prophylaxis starting as early as 14 wk, for those with higher CD4 counts^4^. It is appropriate and timely for India to adopt these guidelines to safeguard future treatment options for HIV-infected women and their children.

We observed a high rate of development of NVP resistance among treated women, similar to observations from other countries with HIV-1 clade C epidemic^12^,^13^. The limitations of our study included the relatively small sample size and the fact that only 20 specimens (of the expected 28) were analyzable. Repeat testing of the women and children over a period of time could have helped understand the natural history of these mutations. Further, the technique used here (population sequencing) may have underestimated the prevalence of mutations compared to more sensitive assays like LigAmp and oligonucleotide ligase assay (OLA). Inspite of these limitations, our findings emphasize the need to implement more effective PPTCT regimens, minimizing emergence of drug resistance and thereby preserving long-term treatment options for HIV-infected women in India.

## References

[1] (2007). UNAIDS WHO. HIV/AIDS Epidemic update. Epidemiological fact sheet on HIV/AIDS, India- WHO/UNAIDS. 2008 update131.

[2] (2006). NACO. Guidelines for HIV care and treatment in infants and children.

[3] Giaquinto C, Rampon O, de Rossi A (2006). Antiretroviral therapy for prevention of mother to child transmission. *Clin Drug Invest*.

[4] (2009). Use of antiretroviral drugs for treating pregnant women and preventing HIV infection in infants. *Rapid Advice, WHO guidelines*.

[5] Jourdain G, Ngo-Giang-Huong N, Le Coeur S, Bowonwatanuwong C, Kantipong P, Leechanachai P (2004). Intrapartum exposure to nevirapine and subsequent maternal responses to nevirapine-based antiretroviral therapy. *N Engl J Med*.

[6] (2004). *ViroSeq HIV-1 Genotyping System v2.0, User manual*.

[7] Johnson VA, Brun-Vezinet F, Clotet B, Gunthard HF, Kuritzkes DR, Pillay D (2008). Update of the drug resistance mutations in HIV-1. *Top HIV Med*.

[8] Bennett DE, Camacho RJ, Otelea D, Kuritzkes DR, Fleury H, Kiuchi M (2009). Drug resistance mutations for surveillance of transmitted HIV-1 drug resistance: 2009 Update. *PLoS One*.

[9] Lakshmi R, Ramesh K, Narayanan PR, Swaminathan S (2009). Antiretroviral drug-resistant mutations at baseline and at time of failure of antiretroviral therapy in HIV type 1-coinfected TB patients. *AIDS Res Hum Retroviruses*.

[10] Lall M, Gupta RM, Sen S, Kapila K, Tripathy SP, Paranjape RS (2008). Profile of primary resistance in HIV-1-infected treatment-naive individuals from Western India. *AIDS Res Hum Retroviruses*.

[11] Balakrishnan P, Kumarasamy N, Kantor R, Solomon S, Vidya S, Mayer KH (2005). HIV type 1 genotypic variation in an antiretroviral treatment-naive population in southern India. *AIDS Res Hum Retroviruses*.

[12] Eshleman SH, Hoover DR, Chen S, Hudelson SE, Guay LA, Mwatha A (2005). Nevirapine resistance in women with HIV-1 subtype C compared to subtype A and D after administration of single dose nevirapine. *J Infect Dis*.

[13] Arrive E, Newell ML, Ekouevi DK, Chaix ML, Thiebaut R, Masquelier B (2007). Prevalence of resistance to nevirapine in mothers and children after single-dose exposure to prevent vertical transmission of HIV-1: a meta-analysis. *Int J Epidemiol*.

[14] Stringer JS, McConnell MS, Kiarie J, Bolu O, Anekthananon T, Jariyasethpong T (2010). Effectiveness of non-nucleoside reverse-transcriptase inhibitor-based antiretroviral therapy in women previously exposed to a single intrapartum dose of nevirapine: A multi-country, prospective cohort study. *PLoS Med*.

[15] Moorthy A, Gupta A, Bhosala R, Tripathy S, Sastry J, Kulkarni S (2009). Nevirapine resistance and breast - milk HIV transmission: Effects of single and extended-dose nevirapine prophylaxis in subtype C HIV-infected infants. *PLos One*.

[16] Eshleman SH, Mracna M, Guay LA, Deseyve M, Cunningham S, Mirochnick M (2001). Selection and fading of resistance mutations in women and infants receiving nevirapine to prevent HIV-1 vertical transmission (HIVNET 012). *AIDS*.

[17] Lidstrom J, Hoover DR, Kumwenda N, Li Q, Mofenson LM, Fowler MG (2010). Comparison of nevirapine (NVP) resistance among HIV infected infants who received extended NVP plus zidovudine prophylaxis vs. extended NVP prophylaxis alone: the PEPI-Malwi study. *Conference on Retrovirus and Opportunistic Infections*.

[18] Chaix M (2005). Addition of 3 days of ZDV+3TC postpartum to a short course of ZDV+3TC and single dose NVP provides low rate of NVP resistance mutations and high efficacy in preventing postpartum HIV-1 transmission. *Conference on Retrovirus and Opportunistic Infections*.

